# Supplementary material for: Detecting bulbar amyotrophic lateral sclerosis (ALS) using automatic acoustic analysis
Source: Biomed Eng Online. 2024 Feb 4;23:15. doi: 10.1186/s12938-023-01174-z (PMC10838438; doi:10.1186/s12938-023-01174-z)
Supplement: Supplementary file 1 — Additional file 1: Table S1. Acoustic features and their descriptions (Winterlight pipeline). [file 12938_2023_1174_MOESM1_ESM.doc]

**Additional file 1: Table S1.** Acoustic features and their descriptions (Winterlight pipeline).

| Feature name | Description |
| --- | --- |
| fundamental_frequency_max | The max of the sequence of fundamental frequency values extracted from the audio file, using the Parselmouth library (equivalent to Praat method for computing fundamental frequency). In Hz. The cutoff range is 70-620 Hz. |
| fundamental_frequency_mean | The mean of the sequence of fundamental frequency values extracted from the audio file, using the Parselmouth library (equivalent to Praat method for computing fundamental frequency). In Hz. The cutoff range is 70-620 Hz. |
| fundamental_frequency_median | The median of the sequence of fundamental frequency values extracted from the audio file, using the Parselmouth library (equivalent to Praat method for computing fundamental frequency). In Hz. The cutoff range is 70-620 Hz. |
| fundamental_frequency_min | The min of the sequence of fundamental frequency values extracted from the audio file, using the Parselmouth library (equivalent to Praat method for computing fundamental frequency). In Hz. The cutoff range is 70-620 Hz. |
| fundamental_frequency_range | The difference between the maximum and minimum fundamental frequency value extracted from the audio file. Computed as (fundamental_frequency_max - fundamental_frequency_min). In Hz. The cutoff range is 70-620 Hz. |
| fundamental_frequency_variance | The variance of the sequence of fundamental frequency values extracted from the audio file, using Praat. In Hz. The cutoff range is 70-620 Hz. |
| hnr_ac_max | The max degree of acoustic periodicity, in dB, using the autocorrelation method. The ratio of energy between the signal in the periodic part and the noise (e.g. An HNR of 0 dB means there is equal energy in the harmonics and the noise). |
| hnr_ac_mean | The mean degree of acoustic periodicity, in dB, using the autocorrelation method. The ratio of energy between the signal in the periodic part and the noise (e.g. An HNR of 0 dB means there is equal energy in the harmonics and the noise). |
| hnr_ac_median | The median degree of acoustic periodicity, in dB, using the autocorrelation method. The ratio of energy between the signal in the periodic part and the noise (e.g. An HNR of 0 dB means there is equal energy in the harmonics and the noise). |
| hnr_ac_min | The min degree of acoustic periodicity, in dB, using the autocorrelation method. The ratio of energy beteween the signal in the periodic part and the noise (e.g. An HNR of 0 dB means there is equal energy in the harmonics and the noise). |
| hnr_ac_range | The range in degree of acoustic periodicity, in dB, using the autocorrelation method. The ratio of energy beteween the signal in the periodic part and the noise (e.g. An HNR of 0 dB means there is equal energy in the harmonics and the noise). |
| hnr_ac_variance | The variance in the degree of acoustic periodicity, in dB, using the autocorrelation method. The ratio of energy beteween the signal in the periodic part and the noise (e.g. An HNR of 0 dB means there is equal energy in the harmonics and the noise). |
| hnr_cc_max | The max degree of acoustic periodicity, in dB, using the cross-correlation method. The ratio of energy beteween the signal in the periodic part and the noise (e.g. An HNR of 0 dB means there is equal energy in the harmonics and the noise). |
| hnr_cc_mean | The mean degree of acoustic periodicity, in dB, using the cross-correlation method. The ratio of energy beteween the signal in the periodic part and the noise (e.g. An HNR of 0 dB means there is equal energy in the harmonics and the noise). |
| hnr_cc_median | The median degree of acoustic periodicity, in dB, using the cross-correlation method. The ratio of energy beteween the signal in the periodic part and the noise (e.g. An HNR of 0 dB means there is equal energy in the harmonics and the noise). |
| hnr_cc_min | The min degree of acoustic periodicity, in dB, using the cross-correlation method. The ratio of energy beteween the signal in the periodic part and the noise (e.g. An HNR of 0 dB means there is equal energy in the harmonics and the noise). |
| hnr_cc_range | The range in degree of acoustic periodicity, in dB, using the cross-correlation method. The ratio of energy beteween the signal in the periodic part and the noise (e.g. An HNR of 0 dB means there is equal energy in the harmonics and the noise). |
| hnr_cc_variance | The variance in the degree of acoustic periodicity, in dB, using the cross-correlation method. The ratio of energy beteween the signal in the periodic part and the noise (e.g. An HNR of 0 dB means there is equal energy in the harmonics and the noise). |
| intensity_max | The maximum of the intensity curve (i.e. perceived loudness), relative to 2*10^-5 Pa (normative auditory threshold for a 1000-Hz sine wave). In dB. |
| intensity_mean_db | The mean of the intensity curve (i.e. perceived loudness), relative to 2*10^-5 Pa (normative auditory threshold for a 1000-Hz sine wave). In dB. |
| intensity_mean_energy | The mean of the intensity curve (i.e. perceived loudness), relative to 2*10^-5 Pa (normative auditory threshold for a 1000-Hz sine wave). In Pa2/s (i.e. the mean power) |
| intensity_mean_sones | The mean of the intensity curve (i.e. perceived loudness), relative to 2*10^-5 Pa (normative auditory threshold for a 1000-Hz sine wave), based on the averaging properties of the human ear. In sones. |
| intensity_median | The median of the intensity curve (i.e. perceived loudness), relative to 2*10^-5 Pa (normative auditory threshold for a 1000-Hz sine wave). In dB. |
| intensity_min | The minimum of the intensity curve (i.e. perceived loudness), relative to 2*10^-5 Pa (normative auditory threshold for a 1000-Hz sine wave). In dB. |
| intensity_range | The difference between the maximum and minimum of the intensity curve (i.e. percieved loudness), relative to 2*10^-5 Pa (normative auditory threshold for a 1000-Hz sine wave). In dB. |
| intensity_variance | The variance of the intensity curve (i.e. perceived loudness), relative to 2*10^-5 Pa (normative auditory threshold for a 1000-Hz sine wave). In dB. |
| jitter_ddp | Average absolute difference between consecutive differences between consecutive periods, divided by the average period. The value is three times RAP. A percentage. For the precise procedure, see: http://www.fon.hum.uva.nl/praat/manual/Voice_2__Jitter.html |
| jitter_local | The average absolute difference between consecutive periods, divided by the average period. A percentage. For the precise procedure, see: http://www.fon.hum.uva.nl/praat/manual/Voice_2__Jitter.html |
| jitter_local_absolute | The average absolute difference between consecutive periods, in seconds. For the precise procedure, see: http://www.fon.hum.uva.nl/praat/manual/Voice_2__Jitter.html |
| jitter_ppq5 | Five-point Period Perturbation Quotient, the average absolute difference between a period and the average of it and its four closest neighbours, divided by the average period. A percentage. For the precise procedure, see: http://www.fon.hum.uva.nl/praat/manual/Voice_2__Jitter.html |
| jitter_rap | Relative Average Perturbation, the average absolute difference between a period and the average of it and its two neighbours, divided by the average period. A percentage. For the precise procedure, see: http://www.fon.hum.uva.nl/praat/manual/Voice_2__Jitter.html |
| long_pause_count_normalized | Number of pauses > 2 sec, divided by the length of audio in sec |
| long_pause_duration | The total duration of pauses (unvoiced segments) > 2 sec, in seconds |
| mean_pause_duration | The duration of unvoiced segments divided by total number unvoiced segments (VAD). Includes all unvoiced segments (including < 150 ms). |
| medium_pause_count_normalized | Number of pauses 1-2 sec, divided by the length of audio in sec |
| medium_pause_duration | The total duration of pauses (unvoiced segments) that are 1-2 sec, in seconds |
| pause_word_ratio | Number of unvoiced segments longer than 150 ms divided by number of voiced segments (comes from VAD module). |
| phonation_rate | Number of voiced samples (50ms windows) over the total number of samples |
| shimmer_apq11 | The 11-point Amplitude Perturbation Quotient, the average absolute difference between the amplitude of a period and the average of the amplitudes of it and its ten closest neighbours, divided by the average amplitude. A percentage. |
| shimmer_apq3 | The three-point Amplitude Perturbation Quotient, the average absolute difference between the amplitude of a period and the average of the amplitudes of its neighbours, divided by the average amplitude. A percentage. |
| shimmer_apq5 | The five-point Amplitude Perturbation Quotient, the average absolute difference between the amplitude of a period and the average of the amplitudes of it and its four closest neighbours, divided by the average amplitude. A percentage. |
| shimmer_dda | The average absolute difference between consecutive differences between the amplitudes of consecutive periods. A percentage. |
| shimmer_local | The average absolute difference between the amplitudes of consecutive periods, divided by the average amplitude. A percentage. |
| shimmer_local_db | The average absolute base-10 logarithm of the difference between the amplitudes of consecutive periods. In dB. |
| short_pause_count_normalized | Number of pauses < 1 sec, divided by the length of audio in sec |
| short_pause_duration | The total duration of pauses (unvoiced segments) < 1 sec, in seconds |
| zcr_kurtosis | Kurtosis of the number of zero crossings (changes from positive to negative or negative to positive signal value) across all voiced frames |
| zcr_mean | Mean number of zero crossings (changes from positive to negative or negative to positive signal value) across all voiced frames |
| zcr_skewness | Skewness of the number of zero crossings (changes from positive to negative or negative to positive signal value) across all voiced frames |
| zcr_var | Variance of the number of zero crossings (changes from positive to negative or negative to positive signal value) across all voiced frames |
| articulation_rate | The total number of syllables for words in the transcript (note: "xxx" unintelligible words are counted as having 1 syllable, based on the average number of word syllables in an internal dataset) divided by the total duration of voiced audio (i.e. speech). In number of syllables per second. The value will be 0 if there are no words/speech in the audio. |
| avg_word_duration | The length of audio (in seconds) divided by the number of words in transcript |
| speech_rate | Number of words per minute |
